# Purification and Characterization of Catalase from Marine Bacterium *Acinetobacter* sp. YS0810

**DOI:** 10.1155/2014/409626

**Published:** 2014-06-18

**Authors:** Xinhua Fu, Wei Wang, Jianhua Hao, Xianglin Zhu, Mi Sun

**Affiliations:** ^1^Key Laboratory of Sustainable Development of Marine Fisheries, Ministry of Agriculture, Yellow Sea Fisheries Research Institute, Chinese Academy of Fishery Sciences, Qingdao 266071, China; ^2^Shanghai Ocean University, Shanghai 201306, China; ^3^Weifang Medical University, Weifang 261053, China; ^4^School of Electrical and Information Engineering, Jiangsu University, Zhenjiang 212013, China; ^5^Marine Products Resource and Enzyme Engineering Laboratory, Yellow Sea Fisheries Research Institute, 106 Nanjing Road, Qingdao, Shandong 266071, China

## Abstract

The catalase from marine bacterium *Acinetobacter* sp. YS0810 (YS0810CAT) was purified and characterized. Consecutive steps were used to achieve the purified enzyme as follows: ethanol precipitation, DEAE Sepharose ion exchange, Superdex 200 gel filtration, and Resource Q ion exchange. The active enzyme consisted of four identical subunits of 57.256 kDa. It showed a Soret peak at 405 nm, indicating the presence of iron protoporphyrin IX. The catalase was not apparently reduced by sodium dithionite but was inhibited by 3-amino-1,2,4-triazole, hydroxylamine hydrochloride, and sodium azide. Peroxidase-like activity was not found with the substrate o-phenylenediamine. So the catalase was determined to be a monofunctional catalase. N-terminal amino acid of the catalase analysis gave the sequence SQDPKKCPVTHLTTE, which showed high degree of homology with those of known catalases from bacteria. The analysis of amino acid sequence of the purified catalase by matrix-assisted laser desorption ionization time-of-flight mass spectrometry showed that it was a new catalase, in spite of its high homology with those of known catalases from other bacteria. The catalase showed high alkali stability and thermostability.

## 1. Introduction

Catalases (hydrogen peroxide: hydrogen peroxide oxidoreductase, EC 1.11.1.6) involve disproportionation of hydrogen peroxide to water and oxygen effectively, widely distributed in nature and found in bacterial, plant, and animal cells. The active enzymes are important members of the cellular defense system protecting the cell from oxidative damage [1, 2]. Cellular metabolism of molecular oxygen results in reactive oxygen species (ROS), such as superoxide anion radical (O_2_^−∙^), hydroxyl radical (OH^∙^), and hydrogen peroxide (H_2_O_2_) [3] in all aerobically grown microorganisms. ROS are highly toxic to cells for they are implicated in damage to many biological macromolecules including proteins, DNA, and membrane lipids [3]. Specific enzyme systems are used to eliminate ROS. Toxic O_2_^−∙^ is dismutated to H_2_O_2_ by superoxide dismutase, and accumulation of toxic H_2_O_2_ is prevented by catalase [2, 4].

Catalases include three families: monofunctional catalases, bifunctional catalases-peroxidases, and Mn catalases. Monofunctional catalases and bifunctional catalases are heme catalases, containing iron-protoporphyrin IX as prosthetic group in their active sites, whereas Mn catalases are nonheme catalases. Catalases catalyze decomposed H_2_O_2_ to water and oxygen, whereas peroxidases are characterized by the oxidation of various organic compounds. Monofunctional catalases, containing four subunits, are composed of two classes based on the size of the subunits: small-subunit catalases (<60 kDa) and large-subunit catalases (>75 kDa) [5].

In recent years, the use of H_2_O_2_ has grown rapidly for sterilization or bleaching processes in some medical, food, and textile operations. The removal of superabundant H_2_O_2_ that persists in products or surroundings by catalases is drawing attention as a substitute for chlorate compounds, which are toxicant and polluting. For this purpose, it is very necessary to produce an economical, highly active, and highly stable catalase. The applied research of catalases promotes the research of purification and biochemical properties of themselves [6–8].

In the previous research, we had screened out a marine strain with high catalase activity. The strain was identified and designated as* Acinetobacter *sp. YS0810 by 16S rDNA sequence (Genbank accession number JX221556). In this study, we have used successive steps of protein precipitation and chromatography to purify the catalase from marine strain* Acinetobacter* sp. YS0810 (YS0810CAT). Here, we have described the characterization of YS0810CAT, including molecular weight, absorption spectra, N-terminal sequence, alkali stability, and thermostability. The results of this study for YS0810CAT lay the foundation for its theoretical research and application in the medical and industrial fields.

## 2. Materials and Methods

### 2.1. Bacterial Strains and Cultivation

The strain* Acinetobacter* sp. YS0810 was isolated from Qingdao coastal, in China. The strain YS0810 was routinely cultivated aerobically in medium [2% (w/v) peptone, 0.2% (w/v) meat extract, 0.2% (w/v) NH_4_Cl, 0.2% (w/v) KH_2_PO_4_, 0.15% (w/v) KH_2_PO_4_] at 220 rpm on a rotary shaker at 28°C for 24 h.

### 2.2. Materials

Acrylamide, methylene-bis-acrylamide, N,N,N′,N′-tetramethylethylenediamine, and ammonium persulfate for sodium dodecyl sulfate polyacrylamide gel electrophoresis (SDS-PAGE) were purchased from Bio-Rad. Blue dextran, thyroglobulin, bovine catalase, bovine serum albumin, and lysozyme were purchased from Sigma. HiPrep DEAE FF 16/10 column, Superdex 200 10/300 GL column, and Resource Q column were purchased from General Electrics. All other chemicals were of the best purity available.

### 2.3. Protein Determination and Enzyme Assays for Catalase Activity

Protein concentrations were determined using Bradford method [9], with bovine serum albumin as the standard. Protein purity was assayed by SDS–PAGE [10]. Enzyme assays for catalase activity were measured on SHIMADZU UV-2550 spectrophotometer equipped with a Peltier-type cell temperature controller. Assays were performed at 25°C in 50 mM Na_2_HPO_4_-NaH_2_PO_4_ buffer (pH 7.0), containing 30 mM H_2_O_2_. Aliquots of enzyme preparation were added to the reaction mixture, and the decrease in absorbance at 240 nm was tested [11]. Extinction coefficient taken as 43.6 (mol/L)^−1 ^ cm^−1^ at 240 nm was used to calculate the specific activity. One unit of catalase activity is defined as the amount of activity required to convert 1 *μ*mol of H_2_O_2_ to water and oxygen per minute at 25°C [12].

### 2.4. Enzyme Purification

YS0810CAT was purified as follows. Cells of 100 mL fermentation broth were collected by centrifugation at the late logarithmic phase, washed two times with 50 mM potassium phosphate buffer (pH 7.0), and suspended in the same buffer. Cells in the suspension were then disrupted by sonic treatment. The suspension was cleared from cell debris by centrifugation at 8000 g for 20 min. After centrifugation, the supernatant was subjected to ethanol fractionation. The fraction corresponding to 30–80% ethanol was collected. Precipitate was redissolved by 50 mM phosphate buffer (pH 7.0).

The fractions were applied to DEAE Sepharose fast flow ion exchange chromatography equilibrated with 50 mM Tris-HCl buffer (pH 7.5) at a flow rate of 1.5 mL/min. The column was washed with the same phosphate buffer (120 mL) and eluted with an increasing step gradient of 15 and 30% (v/v) 1 M sodium chloride in 50 mM Tris-HCl buffer (pH 7.5, 180 mL and 150 mL, resp.). Fractions with high catalase activity eluted at 30% (v/v) 1 M NaCl were pooled, concentrated by ultrafiltration, and applied to Superdex 200 gel filtration chromatography equilibrated with 1 M NaCl in 50 mM potassium phosphate buffer (pH 7.0). Fractions with peak catalase activity were pooled, concentrated by ultrafiltration, and applied to a Resource Q ion exchange chromatography equilibrated with 20 mM Tris-HCl buffer (pH 8.0), washed with the same buffer (10 mL), and eluted with 20 mL of an increasing linear 0 to 1 M NaCl gradient in 20 mM Tris-HCl buffer (pH 8.0). Fractions with peak catalase activity were pooled, concentrated by ultrafiltration, and used as purified enzyme preparation to analyze the characterization of the catalase.

### 2.5. Characterization of the Catalase

The subunit weight of the purified YS0810CAT was tested by electrospray ionization mass spectrometry. The molecular weight of the purified catalase was estimated by Superdex 200 gel filtration chromatography. Molecular weight standards of known molecular weight were blue dextran (2000 kDa), bovine catalase (232 kDa), bovine serum albumin (66.2 kDa), and lysozyme (14.4 kDa).

Absorption spectra (250–800 nm) of the purified catalase were recorded on a SHIMADZU UV-2550 UV-Vis spectrophotometer before and after the addition of 1 mM sodium dithionite at 25°C.

For inhibitors study, enzyme activities of the purified catalase were assayed in the presence of 3-amino-1,2,4-triazole, hydroxylamine hydrochloride, and sodium azide. Effects of the inhibitors on catalase activities were evaluated after incubation of the enzymes for 30 min in 50 mM potassium phosphate buffer (pH 7.0), containing different concentration of the inhibitors. Peroxidase-like activity was determined in 50 mM citric acid buffer (pH 4.5) containing 0.3 mM o-phenylenediamine.

N-terminal sequence of YS0810CAT was analyzed by Edman degradation. The purified enzyme was heated to 95°C for 5 min to denature. The subunits were separated by SDS-PAGE (12% polyacrylamide). The subunit was transferred onto a polyvinylidene fluoride (PVDF) membrane by electrophoretic transfer for N-terminal sequence analysis. Sequence determinations were performed based on the principle of Edman degradation method using Procise491 protein sequencing system of Applied Biosystems.

Amino acid sequence of YS0810CAT was analyzed by matrix-assisted laser desorption ionization time-of-flight (MALDI-TOF) mass spectrometry. Mass spectra were collected on a Voyager-DE PRO from Applied Biosystems.

Catalase activity of YS0810CAT was assayed at various temperatures and in various pH buffer solutions to study its alkali stability and thermostability.

## 3. Results and Discussion

### 3.1. Enzyme Purification

The yield of catalase was 6819 U·mL^−1^ of fermentation broth after incubation of 24 h. Many studies showed that catalase productions by microorganisms did not exceed 5,000 U·mL^−1^ of fermentation broth [7, 13]. Zeng et al. [8] reported that* Serratia marcescens* SYBC08 showed rather high catalase production of 20,289 U·mL^−1^, although Nakayama et al. reported that catalase had a great rising space by adding some suitable inducers such as H_2_O_2_ [12]. Therefore, the yield of the catalase from marine bacterium* Acinetobacter *sp. YS0810 had great potential in application.

**Figure 1:**
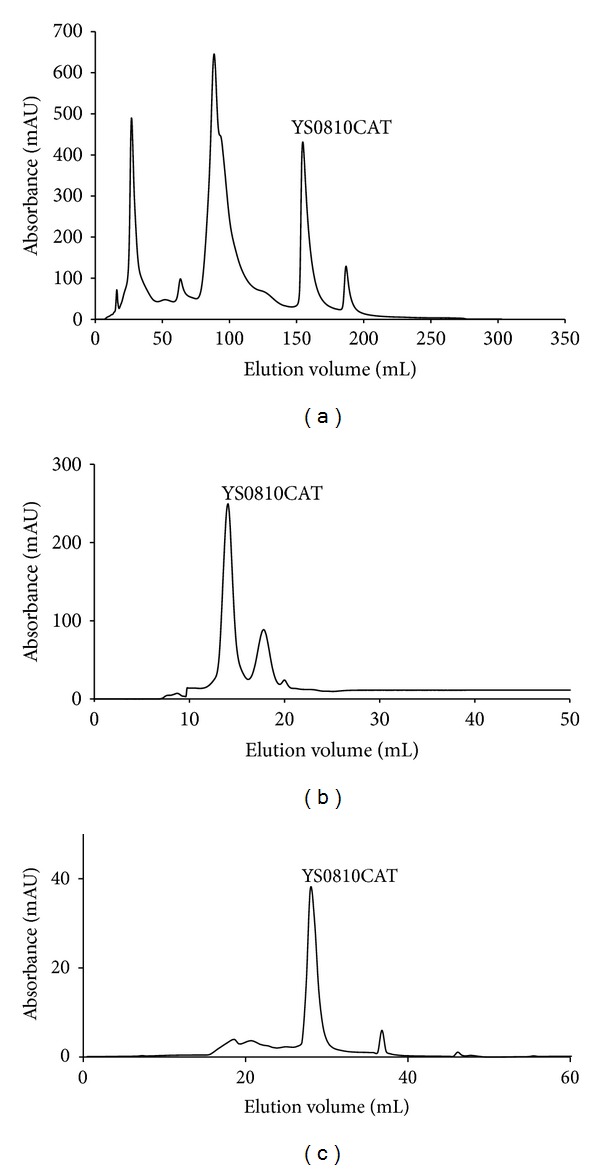
(a) DEAE Sepharose ion exchange chromatography for YS0810CAT; (b) Superdex 200 gel filtration chromatography for YS0810CAT; (c) Resource Q ion exchange chromatography for YS0810CAT.

**Figure 2:**
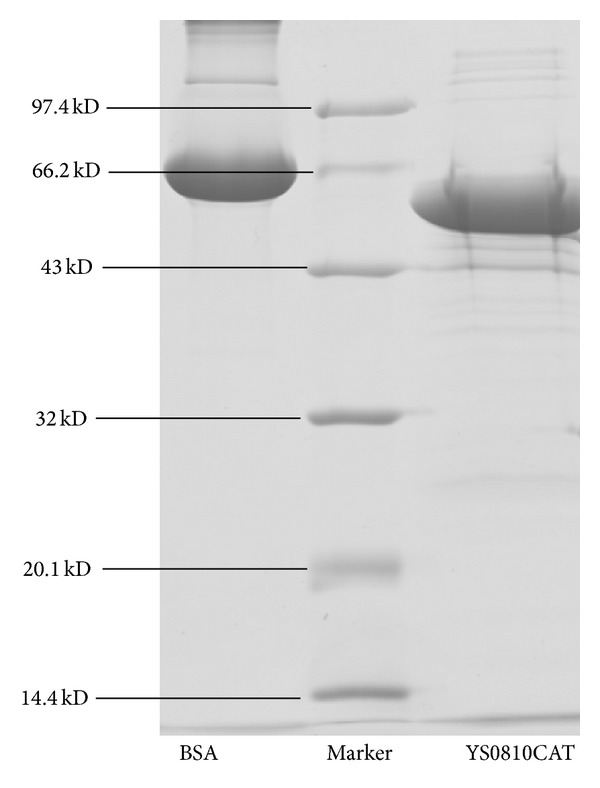
SDS-PAGE for purified YS0810CAT.

The purification procedures of YS0810CAT are summarized in Table 1 and Figure 1. The purification started from crude cell extract containing about 914.38 mg of protein with a specific activity of 745.74 U/mg. By the final step, a 50.04-fold purification was obtained, and 1.26 mg of pure enzyme was obtained with a specific activity of 37318.25 U/mg and a yield of 6.9%. The optimized purification process of the catalase was found to be very effective. There was apparently only a main band on SDS-PAGE gel for the purified enzyme preparation (Figure 2). The molecular weight of a single subunit of the catalase was established as 57 kDa. The 50.04-fold purification achieved in this study was higher than many of reports. Purification of* Thermoascus aurantiacus *M-3 catalase achieved was 5.14-fold [14].

### 3.2. Spectroscopic Characterization

**Figure 3:**
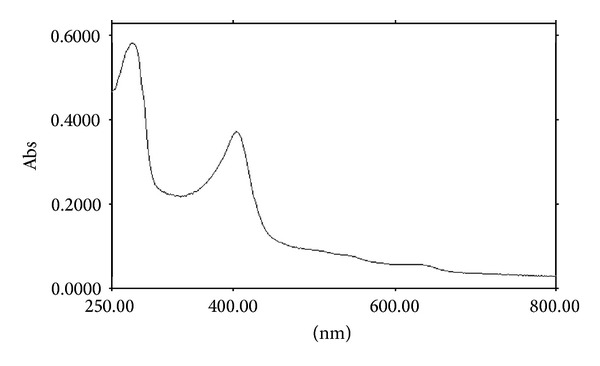
The UV-Vis absorption spectrum of purified YS0810CAT.

**Figure 4:**
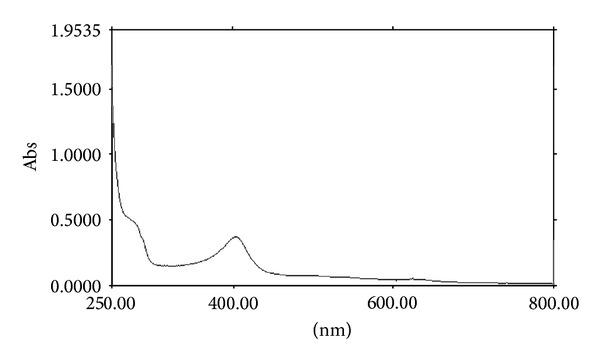
The UV-Vis absorption spectrum of purified YS0810CAT after addition of dithionite.

Absorption spectra of the purified enzyme showed features typical of heme-containing proteins with a Soret band at 405 nm (Figure 3). The A405/A280 ratio of 0.68 was low for heme-containing catalase, which usually exhibited the ratio of approximately 1 [14, 15]. This value might indicate that the heme was partly lost during the purification process. The addition of dithionite did not alter the shape of the spectrum, which suggested that the purified catalase was not reduced by sodium dithionite (Figure 4), a property typical for monofunctional catalases [15, 16].

### 3.3. Sensitivity to Inhibitors

Hydroxylamine hydrochloride and sodium azide are known inhibitors of heme-containing catalase, and 3-amino-1,2,4-triazole is the specific inhibitor of monofunctional catalases. The effects of catalase inhibitors on the catalase activity of the purified enzyme were shown on Table 2. The results of sensitivity of YS0810CAT to inhibitors were similar to those of monofunctional catalases [14–16]. Peroxidase-like activity was not found with the substrate o-phenylenediamine, so the catalase was determined to be a monofunctional catalase.

### 3.4. Molecular Properties

**Figure 5:**
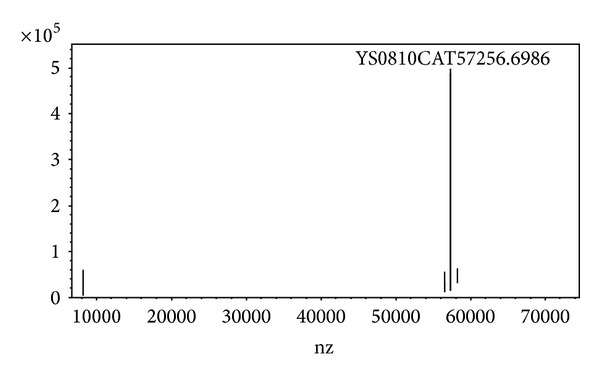
Electrospray ionization mass spectrometry of the purified YS0810CAT.

The size of a single subunit of YS0810CAT was tested as 57.256 kDa by electrospray ionization mass spectrometry (Figure 5), which was in coherence with the result in Figure 2. The total molecular mass of YS0810CAT was determined by an analytical Superdex 200 gel filtration and was estimated at 229 kDa. This indicated that YS0810CAT was tetramer composed of four identical subunits and belonged to small-subunit catalases. The single subunit size and native enzyme molecular mass of this monofunctional catalase were similar to those of bacteria (i.e.,* Vibrio rumoiensis* S-1^T^ with 57.3 kDa and 230 kDa [15],* Serratia marcescens *SYBC08 with 58 kDa and 230 kDa [8], and* Vibrio salmonicida* with 57 kDa and 235 kDa [17], resp.). After the YS0810CAT was transferred to a PVDF membrane, the catalase was subjected to N-terminal amino acid analysis by Edman degradation. Fifteen amino acids were found to be SQDPK KCPVT HLTTE, a sequence that was homologous to those of a few known catalases (*Acinetobacter lwoffii*, Genbank accession number WP_004282247.1,* Acinetobacter* sp. CIP 101966 WP_005265045.1,* Acinetobacter* sp. CIP 102136 WP_005249302.1, Gammaproteobacteria WP_000076364.1,* Citrobacter freundii *WP_003829123.1, and* Pseudomonas fluorescens* AAB17009.1).

The peptides mass fingerprint from MALDI-TOF mass spectrometry was used as a query against the NCBI Protein database (Table 3). Eight peptide sequences were identical with the sequences of the seven catalases from different bacteria, but three peptide sequences did not completely match those of the seven catalases, respectively. Therefore, the YS0810CAT was regarded as a new protein, in spite of homology to a few known catalases.

### 3.5. Alkali Stability and Thermostability

**Figure 6:**
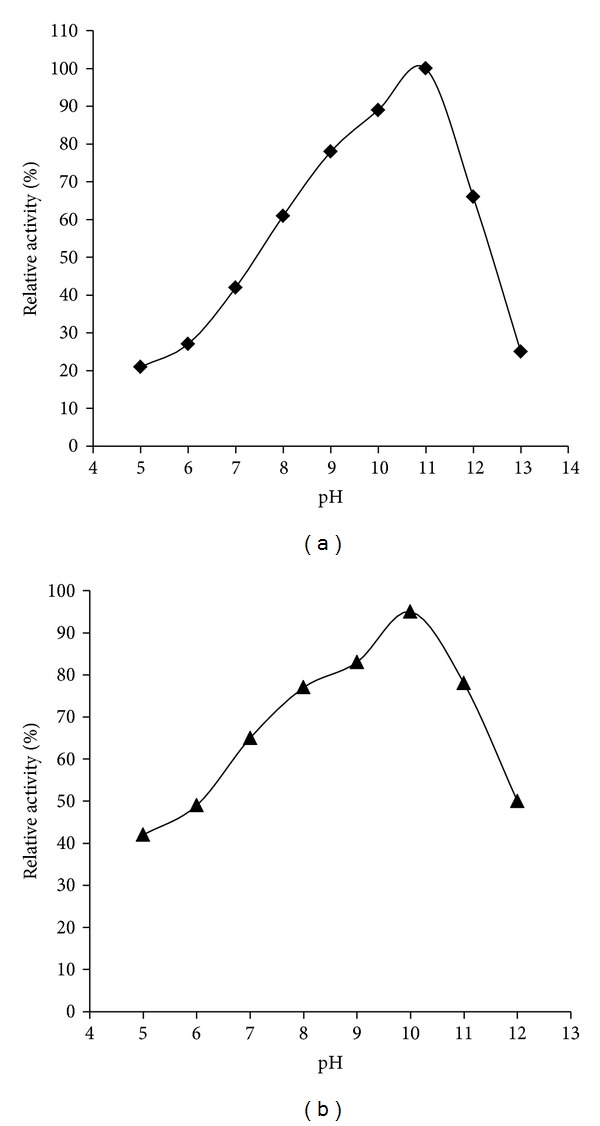
Alkali stability of YS0810CAT. (a) Effect of pH on the activity of YS0810CAT. (b) Effect of pH on the stability of YS0810CAT.

The purified catalase was assayed at 25°C in various pH buffers (pH 5–13). Results (Figure 6(a)) showed that the catalase had an optimal reaction pH at 11. The high enzyme activity was found over a very wide pH range (8.0–12.0). However, the activity rapidly decreased below pH 8.0 or above pH 12, and there was very little activity at pH 5.0. This high enzyme activity pH range was wider than those of monofunctional catalases from* Vibrio salmonicida* [17],* Proteus mirabilis* [17], and* Thermoascus aurantiacus *[14]. The residual activities of catalase were assayed after the purified catalase had been stored at 4°C for 3 h in various pH buffers (pH 5–13), with untreated purified catalase which was 100%. The results showed that the catalase activity was stable at the pH range from 6.0 to 12.0 (Figure 6(b)). The catalase activity retained 95% and 78% of untreated enzyme activity, respectively, after being stored at 4°C for 3 h in pH 10.0 and in pH 11.0. This observation was similar to that obtained from other bacteria [7, 14, 17, 18].

**Figure 7:**
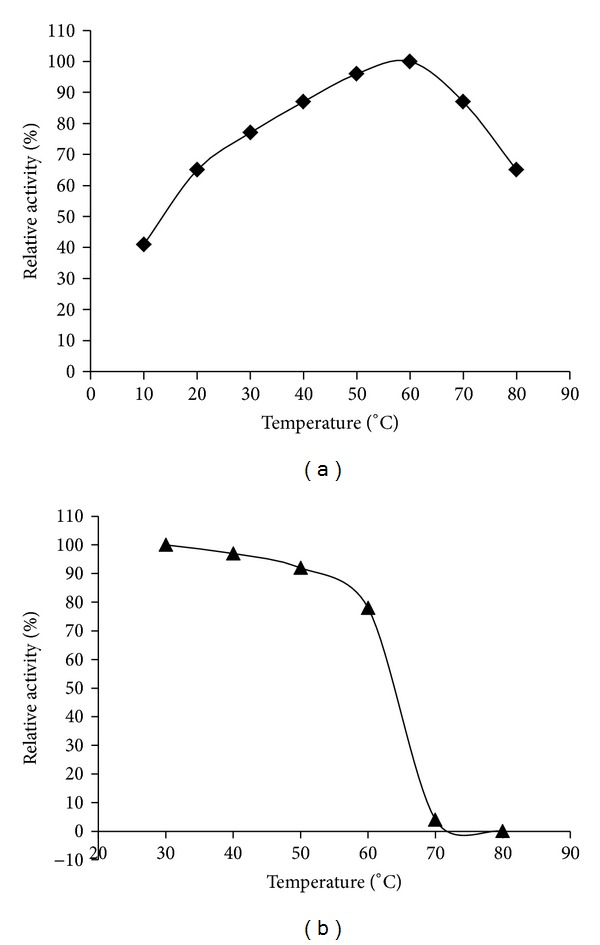
Thermostability of YS0810CAT. (a) Effect of temperature on the activity of YS0810CAT. (b) Effect of temperature on the stability of YS0810CAT.

Effect of the temperature on the activity and stability of the catalase from strain YS0810 was assayed in pH 7.0 buffer. As indicated in Figure 7(a), the catalase had an optimal reaction temperature at 60°C, which was higher than that from other bacteria [7]. The catalase activity from 40 to 70°C was over 80% of the maximal activity at 60°C. When the catalase was incubated for 30 min at temperatures ranging from 10 to 80°C, the activity remained high up to 60°C (Figure 7(b)). The catalase still kept about 78% of its activity at pH 7.0 under 60°C for 30 min. However, an abrupt decline in activity occurred above 60°C. The catalase was almost completely inactivated under 70°C for 30 min. The activities of catalase from* Vibrio salmonicida* and bovine liver were reported to remain about 50% and 40% of maximal activity when incubating at 60°C [7, 19].

Comparison of the enzymatic properties of YS0810CAT with those of other monofunctional catalases from different sources was shown in Table 4.

## 4. Conclusions

In this study, a high activity catalase was obtained by marine bacterium* Acinetobacter *sp. YS0810. The purified catalase was characterized as a monofunctional catalase. Amino acid sequence analyses suggested the YS0810CAT was a new protein, in spite of the conservation of the catalase structures in the microorganisms. The high alkali stability and thermostability of the catalase demonstrated good application prospects in medical and industrial fields.

## Figures and Tables

**Table 1:** Purification of YS0810CAT (100 mL fermentation broth).

Purification steps	Total activity (U)	Total protein (mg)	Specific activity (U/mg)	Yield (%)	Purification (fold)
Cell extracts	681889.741	914.38	745.74	100	1
Ethanol precipitation	616486.03	407.23	1513.85	90.41	2.03
DEAE Sepharose	374847.06	26.68	14049.74	54.97	18.84
Superdex 200	115496.483	4.13	27965.25	16.94	37.50
Resource Q	47021.00	1.26	37318.25	6.9	50.04

**Table 2:** Effect of catalase inhibitors on catalytic activity of YS0810CAT.

Inhibitor	Concentration (mmol/L )	Inhibition (r/%)
NH_2_OH	0.05	78
NaN_3_	0.05	69
3-amino-1,2,4-trizoazole	1	86

**Table 3:** Identification of tryptic peptides of YS0810CAT.

Calculated. Mass	Observed. Mass	±Da	Sequence	Matched bacteria
1447.7765	1447.7192	−0.0573	VGVNHSQVPVNAAR	1, 2, 3, 4
1510.702	1510.6287	−0.0733	ESSQTDLFDAIER	1, 2, 3, 4
1750.8646	1750.8226	−0.042	GDYPLIEVGEFELNR	1, 2, 3, 4, 5
3225.5125	3225.4893	−0.0232	NPENYFQDVEQAAFAPS NLVPGISYSPDR	1, 2, 3, 4
1342.6063	1342.5385	−0.0678	ITGDADFWDFR	1, 2, 3
1385.5604	1385.4937	−0.0667	EDDNDYFSQPR	1, 2, 3, 6, 7
1496.8108	1496.7299	−0.0809	GPLLAQDLWLNEK	1, 2, 3, 4, 5, 6, 7
992.5159	992.457	−0.0589	LVNYADAAR	1, 2, 3, 4

**Table 4:** Comparison of the enzymatic properties of YS0810CAT with those of other monofunctional catalases from different sources.

Property	*Thermoascus aurantiacus *	*Vibrio rumoiensis* S-1^T^	*Vibrio salmonicida*	*Serratia marcescens* SYBC08	*Halomonas *sp. SK1	*Acinetobacter* sp. YS0810
A405/A280 ratio	1.02	0.93	1	0.42	n.d.	0.68
Subunit mass (kD)	75	57	57	58	68	57
Subunits	4	4	4	4	4	4
Optimal pH	6–10	6–10	8.8	7.0–9.0	10	11
Optimal temperature/°C	70	40	0~10	20	n.d.	60
Temperature stability (ResidueActivity)	60°C, 60 min, 100%	60°C, 15 min, 0%	60°C, 20 min, 0%	70°C, 90 min, 57%	55°C, 30 min, 0%	60°C, 30 min, 78%

n. d. means not determined.
